# Dithiocarbamate as a Valuable Scaffold for the Inhibition of Metallo-β-Lactmases

**DOI:** 10.3390/biom9110699

**Published:** 2019-11-05

**Authors:** Ying Ge, Li-Wei Xu, Ya Liu, Le-Yun Sun, Han Gao, Jia-Qi Li, Kewu Yang

**Affiliations:** Key Laboratory of Synthetic and Natural Functional Molecule Chemistry of Ministry of Education, the College of Chemistry and Materials Science, Northwest University, Xi’an 710127, China; geyingxgs@163.com (Y.G.); lwxu717@163.com (L.-W.X.); liuya951214@163.com (Y.L.); marcolsun@163.com (L.-Y.S.); gaohanyfz@163.com (H.G.); lijiaqi01120@163.com (J.-Q.L.)

**Keywords:** antibiotic resistance, metallo-β-lactamase, inhibitor, dithiocarbamate, activity assay

## Abstract

The ‘superbug’ infection caused by metallo-β-lactamases (MβLs) has grown into an emergent health threat. Given the clinical importance of MβLs, a novel scaffold, dithiocarbamate, was constructed. The obtained molecules, DC1, DC8 and DC10, inhibited MβLs NDM-1, VIM-2, IMP-1, ImiS and L1 from all three subclasses, exhibiting an IC_50_ < 26 μM. DC1 was found to be the best inhibitor of ImiS (IC_50_ < 0.22 μM). DC1-2, DC4, DC8 and DC10 restored antimicrobial effects of cefazolin and imipenem against *E. coli*-BL21, producing NDM-1, ImiS or L1, and DC1 showed the best inhibition of *E. coli* cells, expressing the three MβLs, resulting in a 2-16-fold reduction in the minimum inhibitory concentrations (MICs) of both antibiotics. Kinetics and isothermal titration calorimetry (ITC) assays showed that DC1 exhibited a reversible, and partially mixed inhibition, of NDM-1, ImiS and L1, with Ki values of 0.29, 0.14 and 5.06 µM, respectively. Docking studies suggest that the hydroxyl and carbonyl groups of DC1 form coordinate bonds with the Zn (II) ions, in the active center of NDM-1, ImiS and L1, thereby inhibiting the activity of the enzymes. Cytotoxicity assays showed that DC1, DC3, DC7 and DC9 have low toxicity in L929 mouse fibroblastic cells, at a dose of up to 250 μM. These studies revealed that the dithiocarbamate is a valuable scaffold for the development of MβLs inhibitors.

## 1. Introduction

Dating from the discovery of penicillin, β-lactam antibiotics have been developed into the main antibacterial drugs for the treatment of bacterial infection. Currently, about 60% of antibacterial drugs used are β-lactam antibiotics [1]. However, the overuse of β-lactams has resulted in many bacteria that produce β-lactamases, and these bacteria are resistant to the most commonly used antibiotics. β-Lactamases hydrolyse β-lactam antibiotics by breaking the β-lactam ring, rending the drugs ineffective [2]. So far, more than 2000 kinds of β-lactamases have been discovered [3]. They are categorized into four classes:—A, B, C, and D—depending on their amino acid sequence homologies [4]. Class A, C and D enzymes are collectively referred to as serine β-lactamases (SβLs), and catalyze the hydrolysis of β-lactams through a serine residue as an active group. Class B enzymes, also called metallo-β-lactamase (MβLs), are usually dependent on Zn(II) ions [5]. MβLs are further divided into subclasses B1, B2, and B3, based on amino acid sequence homology and Zn(II) content [6].

The “Superbugs” resistance, mediated by MβLs, has resulted in the failure of almost all antibiotics used in clinics, leading to a global medical crisis. Therefore, the development of β-lactamase inhibitors is an essential strategy for maintaining the usefulness of the existing β-lactam antibiotics. Currently, the clavulanic and other SβLs inhibitors, such as sulbactam and tazobactam, have targeted the inactivation of the drug-resistant bacteria that produce SβLs in combination with β-lactam antibiotics [1]. However, these SβL inhibitors are ineffective against the drug-resistant bacteria harboring MβLs [7], and there are no MβL inhibitors for available clinical purposes to date. Due to the biomedical importance of MβLs, a large number of small molecule MβLs inhibitors have been reported [8], such as thiols [9], dicarboxylic acids [10,11], sulfates [12], tetrazoles [13], aspergillomarasmine A (AMA) [14], hydroxamates [15], and ebselen [16].

Dithiocarbamates (DCs) have been reported to be inhibitors of carbonic anhydrase (CA) [17]. CA, like MβLs, are zinc-containing metalloenzymes [18]. Initially, DCs were used to treat crops, to protect against insects and fungi [19]. Later, they were used in anticancer [20,21], antibacterial [22,23], antifungal [24,25], and antiglaucoma [17] applications. Wang and co-workers reported that the DC derivatives with azacycloalkane had an inhibitory effect on drugresistant bacteria containing NDM-1 or IMP-4 genes [26]. Further mechanism studies indicated that these compounds may chelate Zn(II) ions, thereby restoring the antibacterial qualites of meropenem. However, these reported compounds are mostly limited to the aliphatic-substituted dithiocarbamates. In order to develop the structurally diverse dithiocarbamates necessary to screen MβLs inhibitors, we designed and synthesized aromatic acylaminodithiocarbamates and assayed their biological activities.

**Figure 1:**
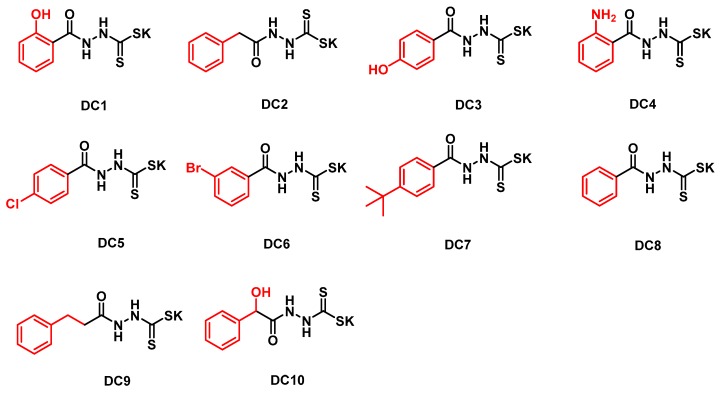
Structures of the synthesized dithiocarbamate derivatives.

To develop the aromatic acylaminodithiocarbamates as inhibitors of MβLs, and to use them in combination with β-lactams to combat antibiotic-resistant bacteria-producing MβLs, in this work, the dithiocarbamates (DC1-10) were synthesized (Figure 1). The positions (ortho, meta, para position) of the aromatic substituents were adjusted, to determine the optimal position for the compound to bind to the active sites of the target enzymes, which confer the best inhibitory effects. The biological activities of these DCs were tested as inhibitors against the purified MβLs VIM-2, IMP-1, NDM-1, ImiS and L1, which are representatives of the B1, B2 and B3 subclasses of MβLs, respectively, and their inhibition mode was evaluated thermodynamically by isothermal titration calorimetry (ITC). Furthermore, the capability of these inhibitors to restore the antimicrobial activity of antibiotics against *E. coli* cells expressing MβLs was determined. In addition, molecular docking was performed, exploring the binding mode between inhibitors and MβLs.

## 2. Materials and Methods

### 2.1. General Information

The substituted benzoic acids were purchased from Aladdin (Shanghai) Trading Co., Ltd. and all other materials were purchased from commercial sources and purified using standard methods. Analytical thin layer chromatography (TLC) was carried out on silica gel F254 plates, with visualization by ultraviolet radiation. ^1^H and ^13^C NMR spectra were recorded on a Bruker 400 MHz NMR spectrometer (Bruker Daltonics Inc., Billerica, MA, USA). Chemical shifts are given in parts per million (ppm) on the delta scale. The peak patterns are reported as singlet (s), doublet (d), triplet (t), quartet (q), and multiplet (m). The spectra were recorded with TMS as an internal standard. Coupling constants (*J*) were reported in Hertz (Hz). Mass spectra were obtained on a micro TOF-Q (BRUKER) mass spectrometer. Inhibition studies were performed on an Agilent 8453 UV-Vis spectrometer (Santa Clara, CA, USA).

### 2.2. Synthesis of Inhibitors

The methyl arylcarboxylates and benzoylhydrazines were prepared using previously reported methods [27]. The intermediate benzoylhydrazine was dissolved in sufficient amount of ethanol and stirred until clarified at room temperature, and the solution was mixed with potassium hydroxide at a molar ratio of 1:1.5. Following this, an equimolar ratio of carbon disulfide was added, and the resulting mixture was stirred at room temperature for 3 h. Insoluble matter was removed by filtration, and the obtained filtrate was distilled under reduced pressure and dried to give the dithiocarbamates. The spectrogram information for the dithiocarbamates (DC1-10) is supplied in the Supporting Information.

### 2.3. Inhibition Studies

The inhibitor concentration causing a 50% decrease in enzyme activity (IC_50_) was determined on an Agilent UV8453 spectrometer at 25 °C using cefazolin as a substrate, for NDM-1, VIM-2, IMP-1 and L1, and imipenem for ImiS, respectively. The dithiocarbamates, MβLs and substrates samples were prepared with 30 mM Tris, pH 7.0. The final concentrations of NDM-1, VIM-2, IMP-1, ImiS, and L1 were 13, 100, 70, 50 and 58 nM, and the concentrations of cefazolin and imipenem were 40 µM, respectively. In the test conditions, the Km for cefazolin (NDM-1, VIM-2, IMP-1 and L1) and imipenem (ImiS) were determined to be 15, 97, 73, 60 and 52 μM, respectively. Enzymes and inhibitors were pre-incubated for 5 min before adding substrate. The hydrolyses of cefazolin and imipenem were monitored at 262 and 300 nm, respectively. The IC_50_ values were nonlinearly fitted by plotting the average percentage inhibition against inhibitor concentration. The inhibition modes of DC1 to ImiS, NDM-1 and L1 were assayed by generating Lineweaver–Burk plots, and *Ki* values were obtained by fitting initial velocity versus substrate concentration at each inhibitor concentration, using SigmaPlot 12.0 (Systat Software Inc., San Jose, CA, USA). Concentrations of DC1 were varied between 0 and 20 μM and substrate (cefazolin and imipenem) were varied between 20 and 120 µM.

### 2.4. Isothermal Titration Calorimetry (ITC) Assays

Isothermal titration calorimetry (ITC) assays were performed on a Malvern MicroCal iTC 200 instrument (Malvern Instruments, Malvern, UK) at 25 °C, by a single injection mode. During ITC experiments the reference cell was filled with degassed water. The MβLs, substrates, and inhibitor DC1 were prepared with 50 mM Tris, pH 7.0. The concentration of MβLs used was in the range 100–200 nM, concentration of substrate was 1 mM, and concentrations of DC1 were varied between 0 and 200 µM. Before starting the measurement, the enzyme was pre-incubated with different concentrations of DC1 for 5 min, and then delivered into the sample cell (210 µL). The hydrolysis reaction in the absence of inhibitors was also determined as a control. The stirring rate of the syringe was set to 750 rpm. The system was equilibrated at the desired 25 °C. Substrate at a concentration of 1 mM (cefazolin for NDM-1 and L1, imipenem for ImiS), in the syringe was titrated into the sample cell, in the mode of a single 38 µL injection. Heat flow was recorded as a function of time. Data were collected every 1 s until the signal reached the baseline and continued to be recorded for the length of time necessary to generate the final baseline.

### 2.5. MIC Determination

The minimum inhibitory concentration (MIC) of antibiotics, both alone and in the presence of enzyme inhibitors, was determined using the broth micro-dilution method. Single colonies of *E. coli* BL21, containing plasmid, pET26b-NDM-1, pET26b-ImiS or pET26b-L1 in lysogeny broth agar plates, were transferred into 5 mL of Mueller–Hinton (MH) liquid medium. Strains grown in MH medium to OD_600_ = 0.45 were used as inocula, after an 84-fold dilution to 1 × 10^5^ CFU/mL in MH medium. Cefazolin and imipenem were dissolved in MH medium to prepare 1024, 512, 256, 128, 64, 32, 16, 8, 4 and 2 μg/mL stock solutions, respectively. Compound DCs as inhibitors were dissolved in MH media, to prepare 1024, 512, 256, 128, 64 and 32 μg/mL stock solutions. The prepared solutions with different antibiotic concentrations (50 μL) were diluted to 100 μL, with different concentrations of inhibitor solution (50 μL), then 100 μL inoculum was added sequentially into the prepared solutions, to yield a quarter of the initial concentrations. The mixtures were incubated at 37 °C for 16 h. The MIC results were taken as the lowest concentration that completely inhibited visible growth. Each measurement was performed in duplicate.

### 2.6. Docking Studies

Docking studies of compound DC1 into the active site of NDM-1 (PDB: 4EYL [28]), CphA (PDB: 2QDS [29], which was used as a closely related relative of ImiS) and L1 (PDB: 2AIO [30]) were performed by AutoDock 4.2 [31]. The carboxyl groups were deprotonated, resulting in an overall charge of -1e for the compounds. A charge of +1.4e was assigned to the two Zn(II) in the active site, while +0.2e was added to each of its ligands [31]. The grid and docking parameter files were prepared using Zn (II) van der Waals parameters = 0.25 kcal/mol and r_0_ = 1.95 Å [32]. Enzymes were treated as a rigid receptor, while ligands were treated as flexible. The grid box was centered between the two active-site Zn (II) ions, with dimensions of 70 × 70 × 70 grid points, with grid points spaced at 0.375 Å. The mutation rate and crossover rates were set at 0.02 and 0.8, respectively, while the maximum energy evaluations and generations’ numbers were set at 2,500,000 and 27,000, respectively. Default values were kept for all other parameters, and no constraints were used. Fifty conformations were generated according to the Lamarckian genetic algorithm, and grouped into clusters based on a root mean square deviation (RMSD) with a tolerance of 2.0 Å. The lowest–energy (highest ranked) clusters were closely examined.

### 2.7. Cytotoxicity Evaluation

The cytotoxicity assays were performed to evaluate the toxicity of DC1, DC3, DC7 and DC9 in mouse fibroblast cells (L-929). The cells were seeded into 96-well plates at a cell density of 1.0 × 10^4^ cells/well, in 100 μL of culture medium and maintained for 24 h. Then, the inhibitors with working concentrations (15.6, 31.25, 62.5, 125, 250, and 500 μM) were added to the 96-well plates and incubated for another 48 h. Three wells containing only cells were suspended in a mixture solution of 98 μL complete medium, as controls for cell viability without inhibitors. Three wells containing only the complete medium were used as blank controls. Following that, the medium was removed, and 100 μL of fresh culture medium and 10 μL MTT were added to each well. After incubation for 4 h, the trays were then vigorously shaken to solubilize the formed product, and the absorbance, at a wavelength of 490 nm, was read on a microplate reader and analyzed. All experiments were conducted in triplicate, and the data are expressed as mean ± standard deviation.

## 3. Results and Discussion

### 3.1. Synthesis

Ten dithiocarbamates (DC1-10) were synthesized by a synthetic route, shown in Scheme A (Supporting Information). Briefly, the intermediate methyl arylcarboxylates were made by esterifying the corresponding benzoic acids, and then were converted into benzoylhydrazines by condensing with hydrazine [27]. The resulting benzoylhydrazines reacted with carbon disulfide in KOH aqueous solution at room temperature for 3 h, and the crude products gained were distilled to offer the dithiocarbamates (DCs) [33]. DCs were characterized by ^1^H and ^13^C NMR and HRMS (Supporting Information).

### 3.2. Inhibition Studies

To evaluate the inhibitory activity of the dithiocarbamates, we over-expressed and purified MβLs NDM-1, VIM-2 and IMP-1 (B1), ImiS (B2), and L1 (B3) from three subclasses as previously reported [34,35,36,37]. The inhibition experiments were performed with an Agilent UV8453 spectrometer. Cefazolin (40 μM) was used as substrate for NDM-1, VIM-2, IMP-1 and L1, and imipenem (40 μM) for ImiS. Hydrolysis of substrates was monitored at 262 (cefazolin) and 300 nm (imipenem). The concentrations of inhibitors were varied between 0 and 200 μM. The initial reaction rates were determined in the absence and presence of inhibitors in triplicate, and the generated average values were recorded.

**Figure 2:**
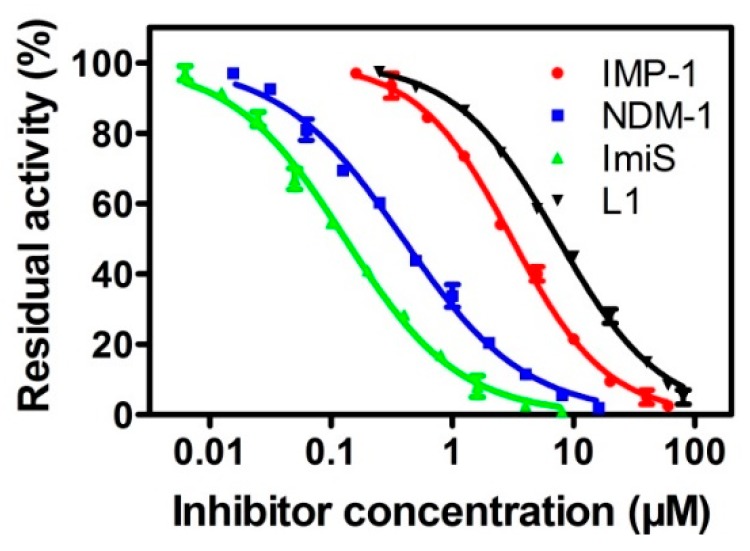
Inhibition curves of DC1 against NDM-1, IMP-1, ImiS and L1.

The collected concentrations of dithiocarbamates DC1-10 causing a 50% decrease in enzyme activity (IC_50_) are listed in Table 1. The results indicate that DC1, DC8 and DC10 were potent broad-spectrum inhibitors of all five MβLs tested, exhibiting IC_50_ values < 26 μM, and DC1 was found to be the most potent inhibitor of ImiS (IC_50_ < 0.22 μM). For NDM-1, DC1-2, DC4, DC6-8, and DC10 showed inhibitory potency with an IC_50_ value range of 0.38–15.25 μM. DC1, DC8, and DC10 inhibited VIM-2 and IMP-1, with an IC_50_ value range of 0.85–9.31 μM, and DC8 was the most potent inhibitor (IC_50_ = 0.85 μM). ImiS was inhibited by DC1-2, DC4, DC6, DC8 and DC10, with an IC_50_ value range of 0.21–2.56 μM. Analysis of IC_50_ data of these DCs reveals an interesting structure–activity trend—the inhibitory activity of a single benzene ring and its ortho-substituted products is better than the avtivity of para- and meta-substituted products. Comparison of the IC_50_ values of DC1 and DC8 indicates that the hydroxyl group had little contribution to the inhibitory activity of DC1. While the inhibition activity of DCs regarding NDM-1 is better than VIM-2, this is probably because the NDM-1 contains a shallower and wider substrate–binding groove, which more easily accommodates substrate or inhibitor molecules [38,39]. The inhibition of DC1 in four MβLs was analyzed, and the generated inhibition curves are shown in Figure 2. It can be observed that the compound had more than 90% inhibition against NDM-1, IMP-1, ImiS, and L1 at concentrations of 20, 10, 40 and 8 μM, respectively. Also, while DC1, DC8 and DC10 were incubated with NDM-1 for 8 h, respectively, the residual activity assays showed that the compounds had no time-dependent inhibition of the enzyme (Figure S1).

**Figure 3:**
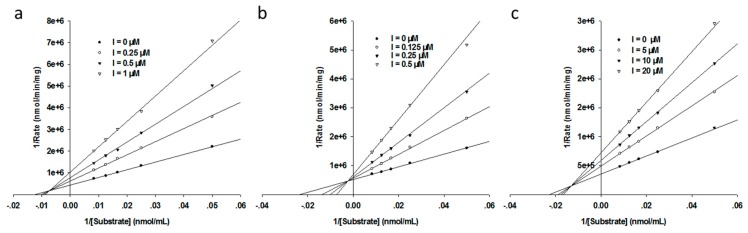
Lineweaver–Burk plots with NDM-1 (**a**), ImiS (**b**), and L1 (**c**) using cefazolin and imipenem as substrate, respectively, in the absence and presence of DC1, at a concentration in the range of 0–20 μM.

To further identify the inhibition mode of the DCs against MβLs, we performed inhibition kinetics of NDM-1, ImiS and L1 by DC1, at different concentrations, with different substrate concentrations. The concentrations of the inhibitor were varied between 0 and 20 μM, and substrate (imipenem or cefazolin) concentrations were varied between 20 and 120 μΜ. Enzyme and inhibitors were pre-incubated for 5 min before starting the kinetic assays. All experimental hydrolytic rates of substrates were determined in triplicate. The inhibition mode of dithiocarbamates on MβLs was identified by generating Lineweaver–Burk plots, and *K*_i_ values were gained by fitting the initial velocity versus substrate concentration at each inhibitor concentration, using SigmaPlot 12.0. The Lineweaver–Burk plots of imipenem and cefazolin hydrolysis by NDM-1, ImiS and L1 in the absence and presence of DC1 are shown in Figure 3. DC1 is shown to exhibit a partially mixed inhibition against NDM-1, ImiS and L1, with *K*_i_ values of 0.29, 0.14 and 5.06 µM, respectively, which were slightly smaller than the corresponding IC_50_ values.

### 3.3. Inhibition Mode Assays

Isothermal titration calorimetry (ITC) was also employed, to identify the inhibition of the dithiocarbamates on MβLs and study their thermodynamic behaviour. The progress of MβL-catalyzed substrate hydrolysis in the absence and presence of inhibitors, was investigated by measuring thermal power (μcal/sec), which is a function of time, on a Malvern MicroCal ITC 200 instrument at 25 °C. The evaluation was performed in 50 mM Tris, pH 7.0. The concentrations of the enzymes NDM-1, ImiS, and L1 were 150, 100 and 100 nM, respectively. The substrate concentration was 1 mM, and its inhibitor was varied between 0 and 200 μM.

**Figure 4:**
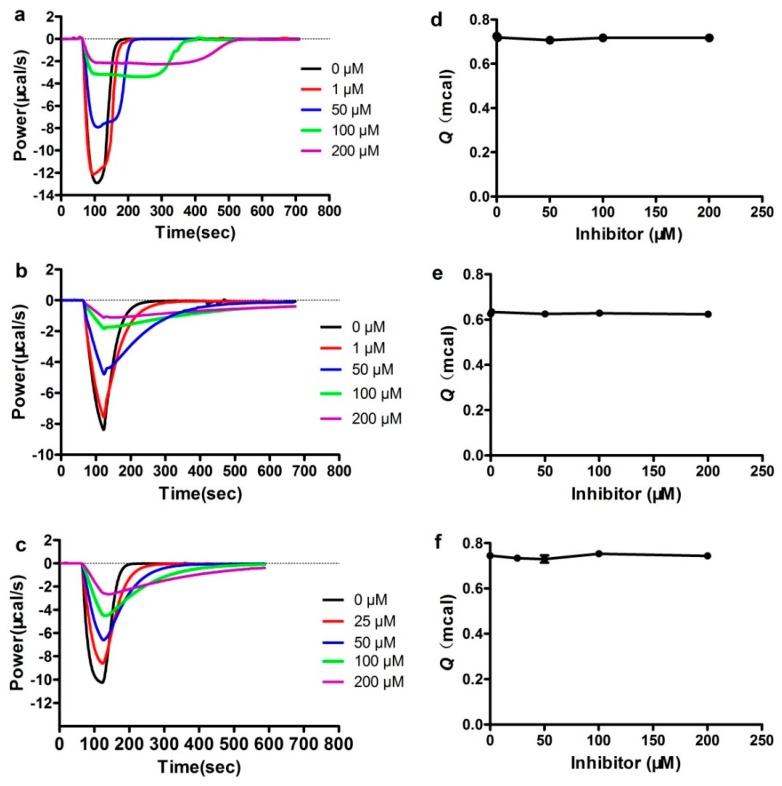
Overlaid heat flow curves of NDM-1- (**a**,**d**) and L1- (**c**,**f**) catalysed hydrolysis of cefazolin and ImiS-catalysed hydrolysis of imipenem (**b**,**e**) in the absence and presence of DC1 at a concentration range of 1–200 µM, using a single injection mode ITC assay at 25 °C.

The heat–flow curves of DC1’s inhibition of the antibiotic hydrolysis catalyzed by NDM-1, ImiS and L1, at different concentrations, are shown in Figure 4a–c. The negative thermal compensation power means that the hydrolysis of substrate was an exothermic reaction. It can be observed that, in the absence of inhibitors, the enzyme activity was higher, the catalytic hydrolysis of substrate emitted more heat per unit time, the corresponding compensation power was higher, the reaction time was shorter, and the heat–flow curve was more concentrated. As the inhibitor concentration increases, the enzyme activity decreases, the heat liberated of substrate per unit time gradually decreases, the time required to hydrolyze the substrate gradually increases, and the heat–flow curve is elongated. ITC assays confirmed the inhibitory effects of the dithiocarbamate on NDM-1, ImiS and L1. However, under the conditions of a certain concentration of enzyme and substrate, the total heat *Q* released (corresponding to the integral area of the heat–flow curve) was a basically fixed value (Figure 4d–f).

Recently, our studies revealed that the *Q* seems to be an important indicator of the inhibitors’ mode of action [40]. When *Q* remained constant, the inhibitor molecule was a reversible inhibitor, but the inhibitor molecule that reduced *Q* was an irreversible inhibitor. It can be observed in Figure 4d–f that the *Q* value remains almost unchanged in the presence or absence of inhibitors, likely because the formation of the enzyme-inhibitor complex (EI), that slowed down substrate hydrolysis, was reversible. ITC assays indicated that the dithiocarbamate which maintains a constant *Q* is a reversible inhibitor. We assayed the binding reversibility of DC1 to MβLs by measuring the enzymatic activity recovery after a large, rapid dilution of the MβL-DC1 complex [41]. The results (Figure S2) suggest that DC1 is a reversible inhibitor; this is consistent with the results from the thermodynamic evaluation.

### 3.4. MIC Assays

The ability of the dithiocarbamate derivatives to inhibit MβLs, and to restore the antimicrobial activity of antibiotics, was investigated by determining the minimum inhibitory concentrations (MICs) of existing antibiotics in the presence and absence of DC1-10, as previously reported [42]. *E. coli* BL21 (DE3) containing plasmids pET26b-NDM-1, pET26b-ImiS and pET26b-L1 were used to assess these inhibitors. The collected MIC data are listed in Table 2A–C. The MIC data show that DC1-2, DC4, DC8 and DC10 gradually restored the antimicrobial effects of the β-lactam antibiotics against NDM-1, ImiS and L1-producing bacteria *E. coli*-BL21, with an increased inhibitor dose. A dose of 64 μg/mL inhibitor resulted in a 2-16-fold decrease in MICs of cefazolin and imipenem, and the DC1 was found to show the best inhibitory effects against the three bacteria tested, resulting in a 16-fold reduction in MICs of both cefazolin and imipenem, indicating that the dithiocarbamate restored activity of the β-lactam antibiotics against the antibiotic–resistant bacteria–producing MβLs. However, the DC3 and DC5-6 had no inhibitory effects on the three bacteria tested. Besides, the inhibitor alone did not inhibit cell growth at a dose up to 512 μg/mL, revealing that the efficacy of dithiocarbamate to restore β-lactam’s antibiotic activity lies in their inhibition of the MβLs harbored in bacteria.

### 3.5. Docking Assays

**Figure 5:**
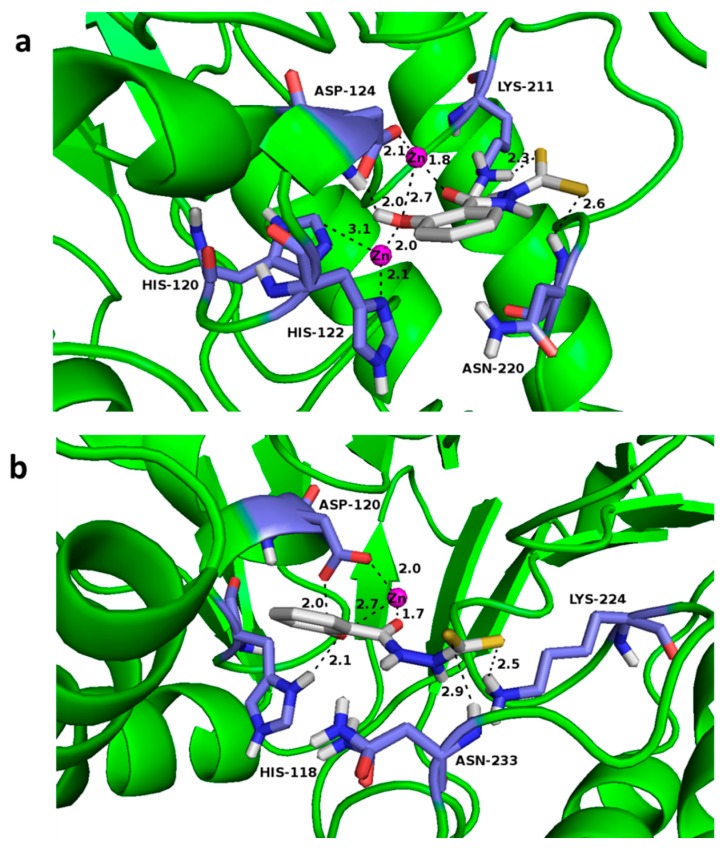

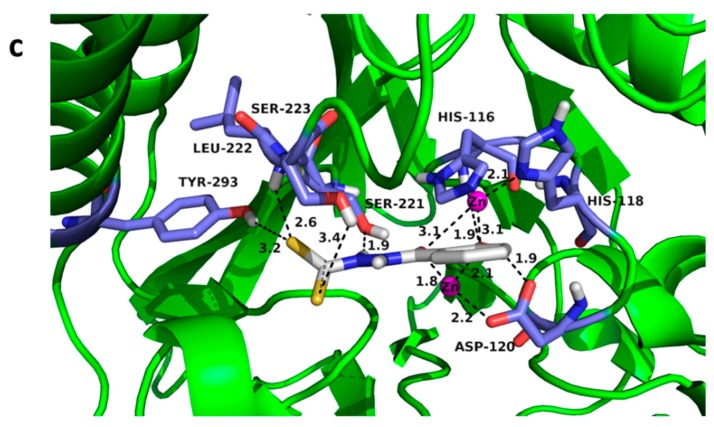
Lowest–energy conformations of DC1, docked into the active site of NDM-1 (PDB code 4EYL) (**a**), CphA (PDB code 2QDS) (**b**) and L1 (PDB code 2AIO) (**c**). Three enzymes were depicted as follows: backbone as cartoon in green; selected residues are shown as sticks, colored by element (H, white; C, skyblue; N, blue; O, red); Zn (II) ions were shown as magenta spheres; compound DC1 was also shown as sticks, with the same color code as amino acid residues, except C in is depicted as white and S as yellow. Characteristic short distances between inhibitors and the protein are indicated by dashed lines. These figures were generated with PyMOL.

To explore the potential binding mode of the dithiocarbamate to MβLs, we docked DC1 into the active sites of NDM-1, CphA (in lieu of ImiS, because the target has no crystal structure yet reported, and shares 96% sequence identity with CphA) and L1. The resulting lowest–energy conformations of those clusters are shown in Figure 5, in which the binding energies are −5.22, −5.99, and −6.64 kcal/mol for the NDM-1/DC1, CphA/DC1 and L1/DC1 complexes, respectively. Figure 5a–c clearly shows that DC1 has a similar binding pattern to NDM-1, CphA, and L1. The hydroxyl oxygen atoms and carbonyl oxygen atoms form coordination bonds with Zn (II) ions of NDM-1, CphA and L1 active sites, and the bond distances are 1.8 and 2.7 Å, 1.7 and 2.7 Å, 1.8 and 2.1 Å, respectively. Further, the hydroxyl oxygen atom in DC1 also forms coordinate bonds and hydrogen bonds with His120_NDM-1_, His118_CphA_ and His116_L1_, in the active centers of NDM-1, CphA and L1, respectively. Moreover, in the complexes NDM-1/DC1, CphA/DC1 and L1/DC1, the hydroxyl hydrogen atom at the benzene ring forms a hydrogen bond with the Asp124_NDM-1_, Asp120_CphA_ and Asp120_L1_, in the active sites of the three enzymes. Also, the sulfur atom in DC1 forms hydrogen bonds with Lys211_NDM-1_, Asn220 _NDM-1_, Lys224_CphA_, Asn233_CphA_, and Ser221_L1_, Leu222_L1_, Tyr293_L1_, in the active centers of NDM-1, CphA and L1, respectively. DC8 was docked into the active sites of NDM-1, CphA and L1, and the results show that it had a similar binding pattern to MβLs as DC1 (Figure S3).

### 3.6. Cytotoxicity Assays

**Figure 6:**
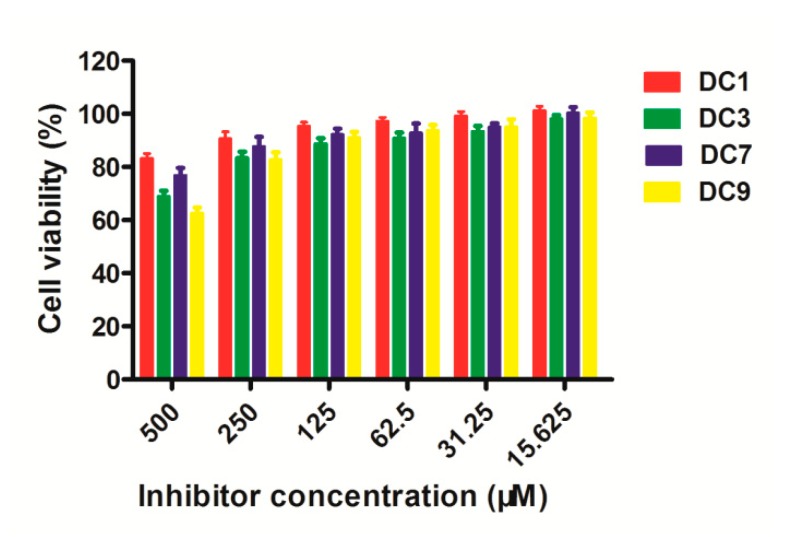
Percent cell viability (relative to viability without compound) of L-929 mouse fibroblastic cells, in the presence of DC1, DC3, DC7 or DC9, at concentrations ranging from 15.6 to 500 µM.

The potential toxicity of dthiocarbamates DC1, DC3, DC7 and DC9 as representatives was assayed using the mouse fibroblast cells (L929) treated with different concentrations of inhibitors (15.625, 31.25, 62.5, 125, 250 and 500 μM). As shown in Figure 6, when the concentration was as high as 250 μM for the tested compounds, the survival rate of L929 mouse cells was higher than 80%, indicating that the dthiocarbamates are basically non-toxic or low-toxic to L929 mouse cells.

## 4. Conclusions

In summary, in response to the enormous biomedical importance of MβLs, ten dithiocarbamate derivatives were synthesized and characterized by ^1^H and ^13^C NMR and HRMS. Bioactivity evaluation showed that the dithiocarbamates DC1, DC8 and DC10 had broad-spectrum inhibition effect on MβLs NDM-1, VIM-2, IMP-1, ImiS, and L1 belonging to three different groups, exhibiting IC_50_ values < 26 μM. DC1 was found to be the best inhibitor of ImiS (IC_50_ < 0.22 μM). MIC assays revealed that DC1-2, DC4, DC8 and DC10 increased antimicrobial effects of cefazolin and imipenem against *E. coli*-BL21–producing NDM-1, ImiS or L1, and DC1 showed the best inhibitory effects on the three bacteria tested, resulting in a 16-fold reduction in the MICs of both antibiotics. Enzyme kinetics and ITC assays indicated that DC1 exhibited a reversible and partially mixed inhibition of NDM-1, ImiS and L1, with *K*_i_ values of 0.29, 0.14, and 5.06 µM, respectively. Docking studies suggest that the hydroxyl and carbonyl groups of DC1 form coordinate bonds with the Zn (II) ions in the active center of NDM-1, ImiS and L1, thereby inhibiting the activity of the enzymes.

## Figures and Tables

**Table 1:** IC_50_ values of dithiocarbamate derivatives (DC1-10) against MβLs (µM).

Compd.	B1			B2	B3
	NDM-1 *^a^*	VIM-2 *^a^*	IMP-1 *^a^*	ImiS *^b^*	L1 *^a^*
DC1	0.38 ± 0.01	9.31 ± 0.20	3.16 ± 0.10	0.21 ± 0.01	7.17 ± 0.10
DC2	5.64 ± 0.10	>200.00	166.22 ± 2.70	2.50 ± 0.10	86.81 ± 1.6
DC3	67.12 ± 0.90	82.76 ± 1.10	>200.00	>200.00	>200.00
DC4	11.39 ± 0.40	139.07 ± 2.05	102.78 ± 1.45	1.08 ± 0.05	176.54 ± 2.80
DC5	91.31 ± 1.70	>200.00	>200.00	128.82 ± 2.10	>200.00
DC6	15.25 ± 0.90	>200.00	>200.00	2.56 ± 0.05	154.37 ± 3.20
DC7	0.52± 0.01	63.46 ± 1.30	96.38 ± 1.20	>200.00	190.79 ± 4.00
DC8	0.53 ± 0.01	3.07 ± 0.10	0.85 ± 0.01	0.67 ± 0.01	25.81 ± 0.12
DC9	62.66 ± 1.05	115.20 ± 3.10	86.16 ± 0.90	156.23 ± 2.20	>200.00
DC10	1.52 ± 0.05	5.25 ± 0.20	2.16 ± 0.05	0.82 ± 0.01	11.68 ± 0.6

The antibiotics used were cefazolin *^a^* and imipenem *^b^*, respectively. The inhibitor concentration used was up to 200 μM.

**Table 2:** Antibacterial activities (MICs, μg/mL) of imipenem on *E. coli* BL21 expressing ImiS (**A**), cefazolin against *E. coli* BL21 expressing NDM-1 (**B**) and L1 (**C**) in the presence of dithiocarbamates at a concentration ranging from 8 to 128 μg/mL and 16 to 256 μg/mL.

**A *^a^***					
Compd.\conc.	8	16	32	64	128
DC1	8	8	4	4	1
DC2	16	8	8	8	4
DC3	16	16	16	16	16
DC4	8	8	8	8	4
DC5	16	16	16	16	16
DC6	16	16	16	16	16
DC7	16	16	16	16	16
DC8	16	8	8	8	8
DC9	16	16	16	16	16
DC10	16	8	8	8	4
**B *^a^***					
Compd.\conc.	8	16	32	64	128
DC1	32	16	16	8	8
DC2	64	64	64	32	32
DC3	128	128	128	128	128
DC4	64	64	32	32	16
DC5	128	128	128	128	128
DC6	128	128	128	128	128
DC7	128	32	16	8	8
DC8	64	64	32	32	16
DC9	128	128	128	128	64
DC10	128	128	128	32	16
**C *^a^***					
Compd.\conc.	16	32	64	128	256
DC1	128	128	64	32	8
DC2	128	128	128	128	64
DC3	128	128	128	128	128
DC4	128	128	128	128	32
DC5	128	128	128	128	128
DC6	128	128	128	128	128
DC7	128	128	128	128	128
DC8	128	128	128	64	32
DC9	128	128	128	128	128
DC10	128	128	128	128	64

*^a^* The MICs of cefazolin alone on *E. coli*-BL21 with NDM-1 or L1 were 128 μg/mL, and the MIC of imipenem alone on *E. coli*-BL21-ImiS was 16 μg/mL.

## Supplementary Materials

The supplementary materials are available online. https://www.mdpi.com/2218-273X/9/11/699/s1.
