# Supplementary material for: Dithiocarbamate as a Valuable Scaffold for the Inhibition of Metallo-β-Lactmases
Source: Biomolecules. 2019 Nov 5;9(11):699. doi: 10.3390/biom9110699 (PMC6920875; doi:10.3390/biom9110699)
Supplement: Supplementary file 1 [file biomolecules-09-00699-s001.pdf]

Supporting Information

Dithiocarbamate as a valid scaffold for the inhibition of metallo-β-lactmases and antibiotic-resistant *E. coli* strains

Table of Contents

Synthetic procedure and NMR and MS characterization of compounds 2
Over-expression and purification of MβLs 4
Time-dependence inhibition experiments 6
Determination of inhibitor reversibility 7
Docking studies 8
References 9

Synthetic procedure and NMR and MS characterization of compounds

Ten dithiocarbamate derivatives were synthesized by the following Scheme A.

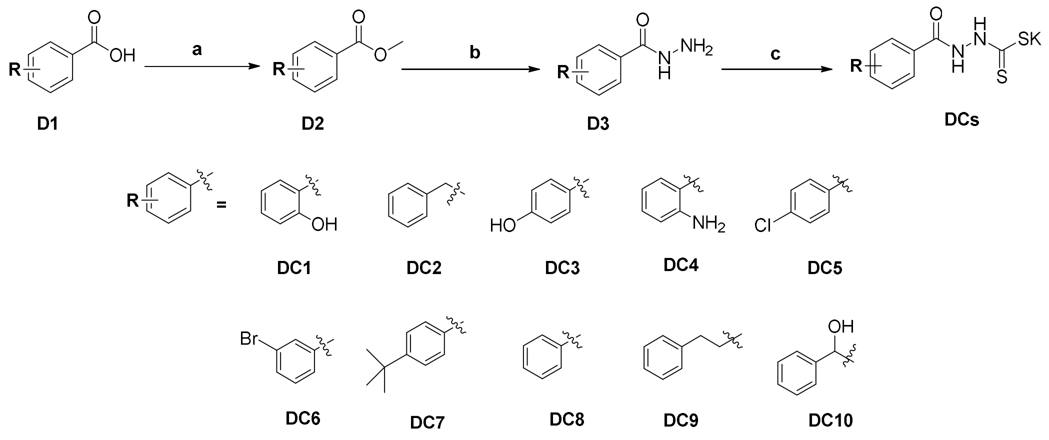

Scheme A. Synthetic route of dithiocarbamate derivatives. The reagents and conditions in the step of dithiocarbamate derivatives synthesis were as follows: (a) CH_3OH, H_2SO_4, 85 °C, 2-6 h; (b) hydrazine hydrate, 100 °C, 12 h; (c) CS_2, KOH, 37 °C, 3 h.

General procedure for preparation of Methyl esters (D2)

The methyl arylcarboxylates (D2) were prepared as previously reported [1]. The correlative substituted benzoic acids (D1, 0.11 mol) were dissolved in 50 mL of methanol, 10 mL of concentrated H_2SO_4 was added, and the system was refluxed at 85 °C for 2-6 h, and the excess methanol was removed under reduced pressure. The reaction mixture was washed with 20% Na_2CO_3 solution to be weakly alkaline, extracted with ethyl acetate 3-4 times, and the organic phase was combined, washed 2-3 times with ultrapure water and saturated brine, and dried on anhydrous Na_2SO_4. The desiccant was removed by filtration under reduced pressure, and the filtrate was concentrated to give D2 as clear oil.

General procedure for preparation of acylhydrazines (D3)

The intermediate benzoylhydrazines (D3) were prepared as previously reported [1]. The methyl ester (D2) dissolved in ethanol were mixed with hydrazine hydrate in a molar ratio of 1:1. The reaction mixture was refluxed at 100 °C for 12 h, and the excess solvent was spun dry, and D3 as white solid was obtained.

General procedure for preparation of dithiocarbamate derivatives

The dithiocarbamates (DCs) were prepared as previously reported [2]. The acylhydrazines (D3) were dissolved in sufficient amount of ethanol and stirred until clarified at room temperature, and the solution was mixed with potassium hydroxide at a molar ratio of 1:1.5, then an equimolar ratio of carbon disulfide was added, and the resulting mixture was stirred at room temperature for 3 h. Insoluble matter was removed by filtration, and the obtained filtrate was distilled under reduced

pressure and dried to give DCs.

Potassium 2-(2-hydroxybenzoyl)hydrazine-1-carbodithioate (DC1)

White solid, yield 76%, m. p: 264 - 265 ℃. ^{1}H NMR (400 MHz, DMSO-*d6*): δ 11.71 (s, 1H), 9.85 (s, 1H), 7.91 (d, *J* = 7.8 Hz, 1H), 7.36 (t, *J* = 7.8 Hz, 1H), 6.98 - 6.90(m, 2H). ^{13}CNMR (101 MHz, DMSO-*d6*): δ 208.15, 159.49, 157.47, 133.40, 130.10, 119.62, 117.21, 116.83. HRMS (ESI) m/z: 226.9943 (Calcd. for $[M-K]^-$: 226.9940 m/z).

Potassium 2-(2-phenylacetyl)hydrazine-1-carbodithioate (DC2)

Orange solid, yield 85%, m. p: 150 - 151 ℃. ^{1}H NMR (400 MHz, DMSO-*d6*): δ 9.27 (s, 1H), 7.48 - 7.07 (m, 5H), 3.94 (s, 2H). ^{13}C NMR (101 MHz, DMSO-*d6*): δ 179.62, 161.87, 136.56, 129.41, 128.63, 127.17, 31.96. HRMS (ESI) m/z: 225.0162 (Calcd. for $[M-K]^-$: 225.0164 m/z)

Potassium 2-(4-hydroxybenzoyl)hydrazine-1-carbodithioate (DC3)

White solid, yield 86%, m. p: 298 - 299 ℃. ^{1}H NMR (400 MHz, DMSO-*d6*): δ 9.97 (s, 1H), 7.95 - 7.23 (m, 2H), 7.23 - 6.70 (m, 2H). ^{13}C NMR (101 MHz, DMSO-*d6*): δ 176.08, 163.95, 158.39, 127.82, 115.73, 115.58. HRMS (ESI) m/z: 226.9943 (Calcd. for $[M-K]^-$: 226.9948 m/z)

Potassium 2-(2-aminobenzoyl)hydrazine-1-carbodithioate (DC4)

Pale solid, yield 72%, m. p: 233 - 234 ℃. ^{1}H NMR (400 MHz, DMSO-*d6*): δ 10.62 (s, 1H), 7.36 (d, *J* = 7.7 Hz, 1H), 7.18 (t, *J* = 7.6 Hz, 1H), 6.74 (d, *J* = 8.2 Hz, 1H), 6.58 (t, *J* = 7.4 Hz, 1H), 6.35 (s, 2H). ^{13}C NMR (101 MHz, DMSO-*d6*): δ 178.23, 169.21, 150.06, 132.09, 127.12, 117.00, 115.70, 115.60. HRMS (ESI) m/z: 226.0103 (Calcd. for $[M-K]^-$: 226.0108 m/z)

Potassium 2-(4-chlorobenzoyl)hydrazine-1-carbodithioate (DC5)

White solid, yield 74%, m. p: >300 ℃. ^{1}H NMR (400 MHz, DMSO-*d6*): δ 7.80 - 7.74 (m, 2H), 7.56 - 7.51 (m, 2H). ^{13}C NMR (101 MHz, DMSO-*d6*): δ 180.70, 160.40, 134.28, 129.61, 126.95, 124.82. HRMS (ESI) m/z: 244.9610 (Calcd. for $[M-K]^-$: 244.9614 m/z)

Potassium 2-(3-bromobenzoyl)hydrazine-1-carbodithioate (DC6)

Yellow solid, yield 80%, m. p: >300 ℃. ^{1}H NMR (400 MHz, DMSO-*d6*): δ 7.88 (t, *J* = 1.8 Hz, 1H), 7.76 (dt, *J* = 7.8, 1.3 Hz, 1H), 7.54 (dt, *J* = 7.5, 2.1 Hz, 1H), 7.45 (t, *J* = 7.9 Hz, 1H). ^{13}C NMR (101 MHz, DMSO-*d6*): δ 177.42, 163.72, 133.81, 130.60, 128.23, 125.08, 124.36, 122.17. HRMS (ESI) m/z: 288.9099 (Calcd. for $[M-K]^-$: 288.9096 m/z)

Potassium 2-(4-(tert-butyl)benzoyl)hydrazine-1-carbodithioate (DC7)

Light yellow solid, yield 82%, m. p: >300 ℃. ^{1}H NMR (400 MHz, DMSO-*d6*): δ 7.72 (d, *J* = 8.2 Hz, 2H), 7.51 (d, *J* = 8.4 Hz, 2H), 1.31 (s, 9H). ^{13}C NMR (101 MHz, DMSO-*d6*): δ 180.00, 161.26, 152.48, 126.22, 125.12, 123.44, 35.02, 31.42. HRMS (ESI) m/z: 267.0620 (Calcd. for $[M-K]^-$: 267.0624 m/z)

Potassium 2-benzoylhydrazine-1-carbodithioate (DC8)

Yellow solid, yield 72%, m. p: 158 - 159 ℃. ^{1}H NMR (400 MHz, DMSO-*d6*): δ 10.79 (s, 1H), 7.87 (dd, *J* = 7.5, 2.0 Hz, 2H), 7.47 (t, *J* = 7.4 Hz, 2H), 7.24 (m, 1H). ^{13}C NMR (101 MHz, DMSO-*d6*): δ 191.93, 161.22, 129.42, 127.07, 126.99, 125.26. HRMS (ESI) m/z: 210.9994 (Calcd. for $[M-K]^-$: 210.9990 m/z)

Potassium 2-(3-phenylpropanoyl)hydrazine-1-carbodithioate (DC9)

Grayish white solid, yield 88%, m. p: 170 - 171 ℃. ^{1}H NMR (400 MHz, DMSO-*d6*): δ 8.97 (s, 1H), 7.41 - 7.00 (m, 5H), 4.16 (s, 2H), 2.81 (t, *J* = 7.8 Hz, 2H), 2.32 (m, 2H). ^{13}C NMR (101 MHz, DMSO-*d6*): δ 179.17, 162.43, 141.06, 128.76, 128.75, 126.53, 32.57, 27.44. HRMS (ESI) m/z: 239.0307 (Calcd. for $[M-K]^-$: 239.0310 m/z)

Potassium 2-(2-hydroxy-2-phenylacetyl)hydrazine-1-carbodithioate (DC10)

Yellow solid, yield 68%, m. p: 264 - 265 ℃. ^{1}H NMR (400 MHz, DMSO-*d6*): δ 7.46 - 7.24 (m, 5H), 6.25 (s, 1H), 5.65 (s, 1H). ^{13}C NMR (101 MHz, DMSO-*d6*): δ 179.83, 164.27, 141.19, 128.56, 127.97, 126.81, 67.21. HRMS (ESI) m/z: 241.0099 (Calcd. for $[M-K]^-$: 241.0103 m/z)

Over-expression and purification of MβLs

NDM-1: NDM-1 was overexpressed and purified as previously described [3]. *E. coli* BL21 (DE3) cells were transformed with the over-expression plasmid, pET26b-NDM-1. A 10 mL overnight culture of these cells in lysogeny broth (LB) was used to inoculate 4 × 1 L of LB medium containing 25 µg/mL kanamycin. The cells were allowed to grow at 37 °C with shaking until the cells reached an optical density at 600 nm of 0.6-0.8. Protein production was induced with 1 mM IPTG, and the cells were shaken at 25 °C for 3 h. The cells were collected by centrifugation (30 min at 8,275 × g) and resuspended in 25 mL of 30 mM Tris, pH 8.0, containing 500 mM NaCl. The cells were lysed by ultrasonication, and the cell debris was separated by centrifugation (30 min at 32,583 × g). The cleared supernatant was dialyzed versus 30 mM Tris, pH 8.0, containing 100 µM $ZnCl_2$ for 36 h at 4 °C, centrifuged (25 min at 32,583 × g) to remove insoluble matter, and loaded onto an equilibrated Q-Sepharose column. Bound proteins were eluted with a 0-500 mM NaCl gradient in 30 mM Tris, pH 8.0, containing 100 µM $ZnCl_2$ at 2 mL/min. Fractions (2 mL) containing NDM-1 were pooled and concentrated with an Amicon ultrafiltration cell equipped with a YM-10 membrane. The crude protein NDM-1 was run through a G75 column and eluted with 30 mM Tris, pH 8.0, containing 200 mM NaCl. Protein purity was ascertained by SDS PAGE and protein concentration was determined using Beer's law and an extinction coefficient of 27,960 M−1cm−1 at 280 nm.

VIM-2: VIM-2 was overexpressed and purified as previously described [4]. *E. coli* BL21 (DE3) cells were transformed with plasmid pET24a-VIM-2. A 10 mL overnight culture of these cells in lysogeny broth (LB) was used to inoculate 4 × 1 L of LB containing 25 µg/mL kanamycin. The cells were allowed to grow at 37 °C with shaking until they reached an optical density at 600 nm of 0.6-0.8. Protein production was induced with 1 mM IPTG, and the cells were shaken at 25 °C overnight. Cells were removed by centrifugation (30 min at 8,275 x g) and the supernatant was concentrated using an Amicon ultrafiltration cell equipped with an YM-10 membrane. The concentrated protein was dialyzed versus 30 mM Tris, pH 7.6, containing 100 µM $ZnCl_2$ for 4 h,

three times at 4 °C, centrifuged (25 min at 32,583 x g) to remove insoluble matter, and loaded onto an equilibrated Q-Sepharose column. Bound proteins were eluted with a 0-500 mM NaCl gradient in 30 mM Tris, pH 7.6, containing 100 μM $ZnCl_2$ at 2 mL/min. Fractions (2 mL) containing VIM-2 were pooled and concentrated with an Amicon ultrafiltration cell equipped with an YM-10 membrane. The crude protein VIM-2 was run through a G75 column and eluted with 30 mM Tris, pH 7.6, containing 200 mM NaCl. Protein purity was ascertained by SDS-PAGE and protein concentration was determined 8 using Beer's law and an extinction coefficient of 39,000 M-1cm-1 at 280 nm.

IMP-1: IMP-1 was overexpressed and purified as previously described [5]. *E. coli* BL21 (DE3) cells were first transformed with the over-expression plasmid pET26b-IMP-1 and the cells were plated on LB-agar plates containing 25 μg/mL kanamycin. A single colony was used to inoculate 50 mL of LB containing 25 μg/mL kanamycin. After the preculture grew overnight at 37 °C, 10 mL overnight culture of these cells in LB was used to inoculate 4 × 1 L of LB containing 25 μg/mL kanamycin. The cells were allowed to grow at 37 °C with shaking until the cells reached an optical density at 600 nm of 0.6-0.8. Protein production was induced with 1 mM IPTG and 50 μM ZnSO4, and the cultures were allowed to shake at 22° C for 20 h. The cells were collected by centrifugation (30 min at 8,275 × g) and resuspended in 25 mL of 50 mM HEPES, pH 7.5, containing 500 mM NaCl. The cells were lysed by ultrasonication, and the cell debris was separated by centrifugation (30 min at 32,583 × g). The supernatant was dialyzed versus 1 L of 50 mM HEPES, pH 7.5, overnight at 4 °C, centrifuged (25 min at 32,583 × g) to remove insoluble matter, and loaded onto an SP-Sepharose column pre-equilibrated with 50 mM HEPES, pH 7.5, at 2 mL/min. Fractions (2 mL) containing S7 IMP-1 were pooled and concentrated with an Amicon ultrafiltration cell equipped with a YM-10 membrane. The crude protein IMP-1 was run through a G75 column and eluted with 50 mM HEPES, pH 7.5, containing 200 mM NaCl. Protein purity was ascertained by SDS PAGE and protein concentration was determined using Beer's law and an extinction coefficient of 49,000 M-1cm-1 at 280 nm.

ImiS: ImiS was overexpressed and purified as previously described [6]. *E. coli* BL21 (DE3) cells were first transformed with the over-expression plasmid pET-26b-ImiS and the cells were plated on LB-agar plates containing 25 μg/mL kanamycin. A single colony was used to inoculate 50 mL of LB containing 25 μg/mL kanamycin. After the preculture grew overnight at 37 °C, 10 mL overnight culture of these cells in LB was used to inoculate 4 × 1 L of LB containing 25 μg/mL kanamycin. The cells were allowed to grow at 37 °C with shaking until the cells reached an optical density at 600 nm of 0.6-0.8. Protein production was induced with 1 mM IPTG, and the cells were shaken at 25 °C for 3 h. The cells were collected by centrifugation (30 min at 8,275 × g) and resuspended in 25 mL of 30 mM Tris, pH 7.0, containing 500 mM NaCl. The cells were lysed by ultrasonication, and the cell debris was separated by centrifugation (30 min at 32,583 × g). The cleared supernatant was dialyzed versus 30 mM Tris, pH 7.0, containing 100 μM $ZnCl_2$ for 36 h at 4 °C, cen-trifuged (25 min at 32,583 × g) to remove insoluble matter, and loaded onto an equilibrated SP-Sepharose column. Bound proteins were eluted with a 0-500 mM NaCl gradient in 30 mM Tris, pH 7, containing 100 μM $ZnCl_2$, at 2 mL/min. Fractions (2 mL) containing ImiS were pooled and concentrated with an Amicon ultrafiltration cell equipped with a YM-10 membrane. The crude protein ImiS was run through a G75 column and eluted with 30 mM Tris,

pH 7.0, 5 containing 200 mM NaCl. Protein purity was ascertained by SDS PAGE and protein concentration was determined using Beer's law and an extinction coefficient of 37,250 M-1cm-1 at 280 nm.

L1: L1 was overexpressed and purified as previously described [7]. *E. coli* BL21 (DE3) cells were first transformed with the over-expression plasmid pET-26b-L1 and the cells were plated on LB-agar plates containing 25 μg/mL kanamycin. A single colony was used to inoculate 50 mL of LB containing 25 μg/mL kanamycin. After the preculture grew overnight at 37 °C, 10 mL overnight culture of these cells in LB was used to inoculate 4 × 1 L of LB containing 25 μg/mL kanamycin. The cells were allowed to grow at 37 °C with shaking until the cells reached an optical density at 600 nm of 0.6-0.8. Protein production was induced with 1 mM IPTG, and the cells were shaken at 37 °C for 3 h. The cells were collected by centrifugation (30 min at 8,275 × g) and resuspended in 25 mL of 30 mM Tris, pH 8.5, containing 500 mM NaCl. The cells were lysed by ultrasonication, and the cell debris was separated by centrifugation (30 min at 32,583 × g). The cleared supernatant was dialyzed versus 30 mM Tris, pH 8.5, containing 100 μM $ZnCl_2$ for 36 h at 4 °C, cen-trifuged (25 min at 32,583 × g) to remove insoluble matter, and loaded onto an equilibrated Q-Sepharose column. Bound proteins were eluted with a 0-500 mM NaCl gradient in 30 mM Tris, pH 8.5, containing 100 μM $ZnCl_2$ at 2 mL/min. Fractions (2 mL) containing L1 were pooled and concentrated with an Amicon ultra-filtration cell equipped with a YM-10 membrane. The crude protein L1 was run through a G75 column and eluted with 30 mM Tris, pH 8.5, containing 200 mM NaCl. Protein purity was ascertained by SDS PAGE and protein concentration was determined using Beer's law and an extinction coefficient of 54,614 M-1cm-1 at 280 nm.

Time-dependence inhibition experiments

NDM-1 was incubated at 13 nM (the concentration required for the activity assay) with DC1, DC8 and DC10 at 0.38, 0.53 and 1.52 μM (the determined IC_{50} values) for 8 h, respectively. The residual activity assays showed that the compounds had no time-dependent inhibition on the enzyme (Figure S1).

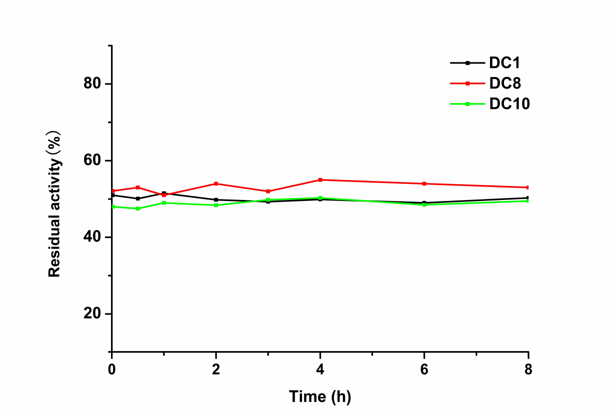

Figure S1. The residual activity of NDM-1 with DC1, DC8 or DC10 at different pre-incubated time.

Determination of inhibitor reversibility

NDM-1, ImiS and L1 were incubated at a concentration of 1.3, 5.0 and 5.8 μM, which is 100-fold over the concentration required for the activity assay, with DC1 at a concentration of 1.5, 0.7 and 25.3 μM (about 5-fold the *Ki* values), respectively. After 10 minutes pre-incubation, the recovery of enzymatic activity was determined after rapid and large dilution (100-fold) with the reaction buffer (30 mM Tris, pH 7.0) containing the substrate cefazolin and imienem at 40 μM (Figure S2). DC1 behave as rapidly reversible inhibitors.

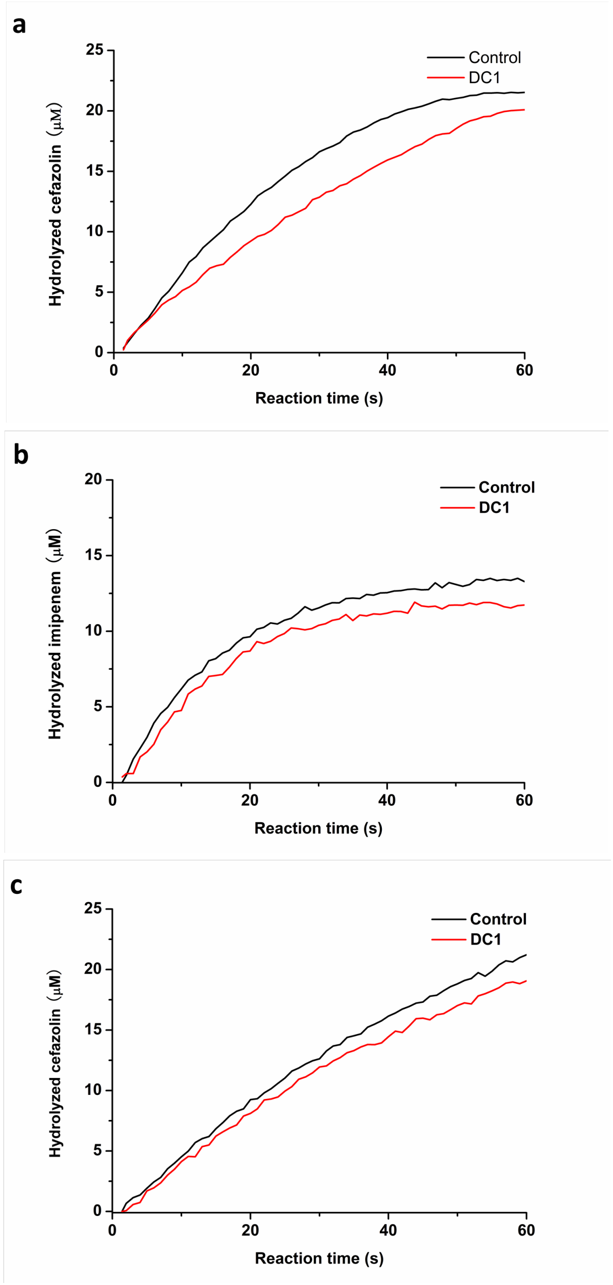

Figure S2. Recovery of NDM-1 (a), ImiS (b) and L1 (c) activity after rapid dilution of the enzyme-inhibitor complex. The curve in black refers to the control sample pre-incubated and diluted in the absence of inhibitor. Curves in red refer to MβLs pre-incubated with DC1.

Docking studies

Docking studies of compound DC8 into the active site of NDM-1 (PDB: 4EYL), CphA (PDB: 2QDS) and L1 (PDB: 2AIO) were performed by AutoDock 4.2. The resulting lowest-energy conformations of those clusters are shown in Figure S3, in which the binding energies are of -4.92, -5.85, and -5.51 kcal/mol for the NDM-1/DC8, CphA/DC8 and L1/DC8 complexes, respectively. Figure S3a-c clearly show that DC8 has a similar binding pattern to NDM-1, CphA, and L1. The carbonyl oxygen atoms form coordination bonds with Zn (Ⅱ) ions at active sites of NDM-1, CphA and L1, and the bond distances are 2.4 and 1.9 Å, 1.7 Å, and 1.9 and 1.9 Å, respectively. Further, the sulfur atom in DC8 forms hydrogen bonds with Asp124NDM-1, Asn233CphA, and His121L1 in the active centers of NDM-1, CphA and L1, respectively.

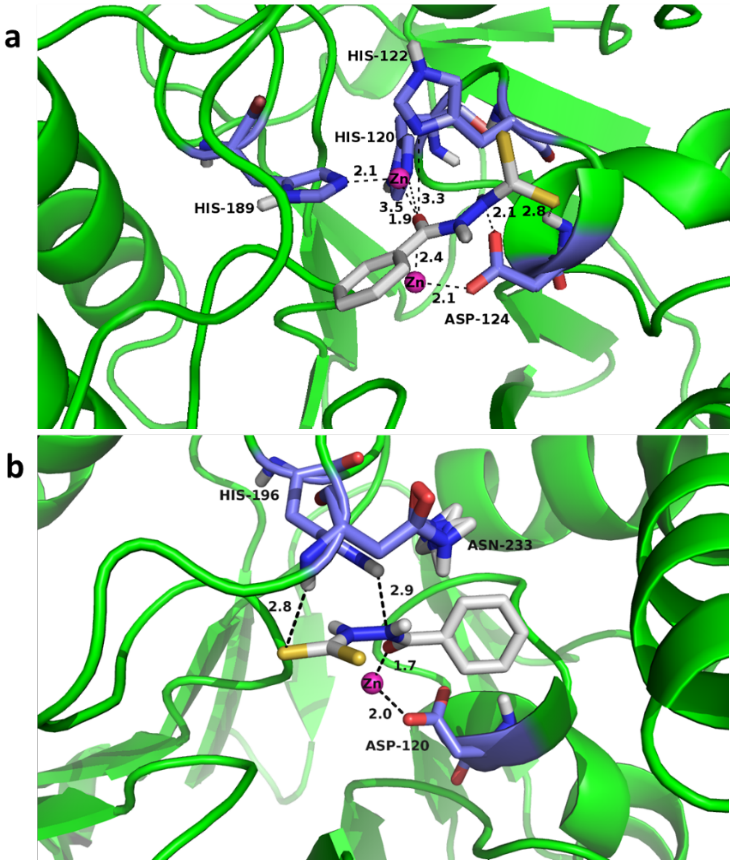

c

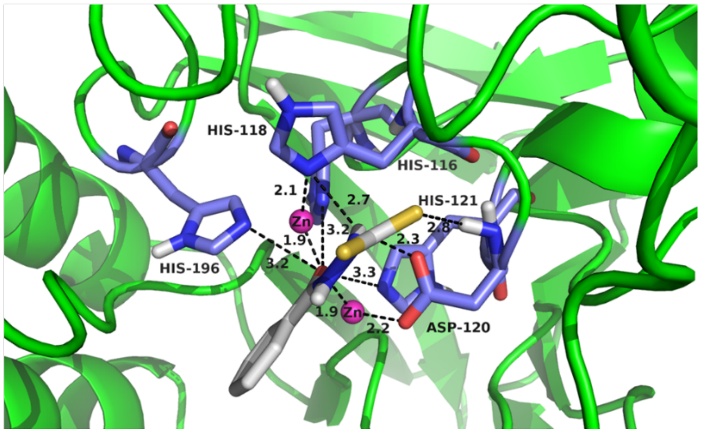

Figure S3. Lowest-energy conformations of DC8 docked into the active site of NDM-1 (PDB code 4EYL) (a), CphA (PDB code 2QDS) (b) and L1 (PDB code 2AIO) (c). Three enzymes were depicted as follows: backbone as cartoon in green and selected residues are shown as sticks colored by element (H, white; C, skyblue; N, blue; O, red), Zn (II) ions were shown as magenta spheres; DC8 was also shown as sticks with the same color code as amino acid residues except C in white and S in yellow. Characteristic short distances between inhibitors and the protein are indicated by dashed lines. These figures were generated with PyMOL.

References

1 Zhai, L.; Zhang, Y. L.; Kang, J. S.; Oelschlaeger, P.; Xiao, L.; Nie, S. S. and Yang, K. W. Triazolylthioacetamide: A Valid Scaffold for the Development of New Delhi Metallo-beta-Lactmase-1 (NDM-1) Inhibitors. *ACS Med Chem Lett.* **2016,** *7*, 413-417.

2 Xiang, Y.; Chen, C.; Wang, W. M.; Xu, L. W.; Yang, K. W.; Oelschlaeger, P. and He, Y. Rhodanine as a Potent Scaffold for the Development of Broad-Spectrum Metallo-beta-lactamase Inhibitors. *ACS Med Chem Lett.* **2018,** *9*, 359-364.

3 Yang, H.; Aitha, M.; Hetrick, A. M.; Richmond, T. K.; Tierney, D. L. and Crowder, M. W. Mechanistic and Spectroscopic Studies of Metallo-β-lactamase NDM-1. *Biochemistry.* **2012,** *51*, 3839-3847.

4 Aitha, M.; Marts, A. R.; Bergstrom, A.; Moller, A. J.; Moritz, L.; Nix, J. C.; Bonomo, R. A.; Page, R. C. and Tierney, D. L. Biochemical, Mechanistic, and Spectroscopic Characterization of Metallo-β-lactamase VIM-2. *Biochemistry.* **2014,** *53*, 7321.

5 Griffin, D. H.; Richmond, T. K.; Sanchez, C.; Moller, A. J.; Breece, R. M.; Tierney, D. L.; Bennett, B. and Crowder, M. W. Structural and kinetic studies on metallo-beta-lactamase IMP-1. *Biochemistry.* **2011,** *50*, 9125-9134.

6 Crawford, P. A.; Sharma, N.; Chandrasekar, S.; Sigdel, T.; Walsh, T. R.; Spencer, J. and Crowder, M. W. Over-expression, purification, and characterization of metallo-beta-lactamase ImiS from Aeromonas veronii bv. sobria. *Protein Expression & Purification.* **2004,** *36*, 272-279.

7 Crowder, M. W.; Walsh, T. R.; Banovic, L.; Pettit, M. and Spencer, J. Overexpression, Purification, and Characterization of the Cloned Metallo-β-Lactamase L1 fromStenotrophomonas maltophilia. *Antimicrobial Agents & Chemotherapy.* **1998,** *42*, 921.
